# Development of Multiplexed Infectious Disease Lateral Flow Assays: Challenges and Opportunities

**DOI:** 10.3390/diagnostics7030051

**Published:** 2017-09-07

**Authors:** Khayriyyah Mohd Hanafiah, Norsyahida Arifin, Yazmin Bustami, Rahmah Noordin, Mary Garcia, David Anderson

**Affiliations:** 1 School of Biological Sciences, Universiti Sains Malaysia, Penang 11800, Malaysia; ybustami@usm.my; 2 Life Sciences, Macfarlane Burnet Institute, Melbourne 3004, Australia; mary.garcia@burnet.edu.au (M.G.); david.anderson@burnet.edu.au (D.A.); 3 Institute for Research in Molecular Medicine, Universiti Sains Malaysia, Penang 11800, Malaysia; syahida_arifin@usm.my (N.A.); rahmah8485@gmail.com (R.N.)

**Keywords:** lateral flow assay, point-of-care, multiplex, diagnostic test, infectious disease, resource-limited settings

## Abstract

Lateral flow assays (LFAs) are the mainstay of rapid point-of-care diagnostics, with the potential to enable early case management and transform the epidemiology of infectious disease. However, most LFAs only detect single biomarkers. Recognizing the complex nature of human disease, overlapping symptoms and states of co-infections, there is increasing demand for multiplexed systems that can detect multiple biomarkers simultaneously. Due to innate limitations in the design of traditional membrane-based LFAs, multiplexing is arguably limited to a small number of biomarkers. Here, we summarize the need for multiplexed LFA, key technical and operational challenges for multiplexing, inherent in the design and production of multiplexed LFAs, as well as emerging enabling technologies that may be able to address these challenges. We further identify important areas for research in efforts towards developing multiplexed LFAs for more impactful diagnosis of infectious diseases.

## 1. Needs and Impetus for Multiplexed Lateral Flow

In resource-constrained settings where lack of access to laboratories constitutes a critical gap in delivering healthcare services, point-of-care (POC) rapid diagnostics adopted with appropriate linkage to health systems have led to faster test-to-result turnaround time, reducing loss to follow up and enabling prompt case management [1]. Among the most familiar of POC formats are lateral flow immunoassays (LFAs), which are typically qualitative diagnostic tests to determine the presence or absence of a target analyte within a non-invasive sample such as whole blood or urine [2].

However, in such settings where lateral flow POCs have prevailing advantages over more sophisticated but laboratory-confined diagnostics, infectious diseases of concern such as tuberculosis, malaria, and parasitic helminthes may share similar symptoms with viral and/or bacterial illnesses, and an individual patient may harbor several co-infections [3]. As such, some public health strategies have often been criticized for being too disease-specific, and not necessarily addressing the overall health of the individual. Indeed, sustainable control strategies, including disease elimination, are likely to require integrated approaches and enabling diagnostic tools that break away from single-disease–single-test approaches that do not account for overall health needs of people living in resource-constrained settings [4]. Although the current evidence base is sparse, such tools arguably have increased impetus for scale-up due to wider market relevance and potential cost-effectiveness. However, more importantly, tests that are able to diagnose or rule out key infections within one interaction at POC may facilitate more pertinent management of the frequently complex nature of patient morbidity. Given economic constraints faced by health programs worldwide, as well as increasing demands for decentralized health care, such tests would arguably be attractive even in higher income settings.

Improved case management of diseases with common symptoms is especially crucial in areas with dense populations where explosive epidemics may occur (such as refugee camps) [5], for prompt discrimination of an endemic infectious disease versus the beginning of an epidemic. The West African Ebola virus outbreak in 2014 was notably difficult for healthcare workers to identify in its early stages due to confusion with malaria, shigellosis, and salmonellosis [6], with higher patient deaths associated with longer referral pathway [7].

Additionally, some infections for which active infection has been difficult to screen serologically such as tuberculosis may require the detection of multiple biomarkers such as combining detection of antigen-specific antibodies and antigens to reach acceptable levels of sensitivity [8,9].

Arising from these needs, the demand for multiplexed diagnostics has become more pressing. Microfluidic biosensors are a natural platform for multiplexed diagnostics due to the minute size of micro-systems and microarray capacity [10]. Indeed, there have been several reports of various tests able to detect multiple analytes on lab-on-a-chip devices with high accuracy [11]. However, these successes in the laboratory are yet to be scalable to reach end-user market due to challenges in mass production, particularly sensor fabrication costs, which limits their translation into POC tests. Given the demand for multiplexed testing and prevailing advantages of the LFA over alternative rapid diagnostic platforms for resource-constrained settings [12], this article aims to address the question of whether the so-called simplicity of membrane-based LFA may be adaptable to multiplexed formats.

## 2. Challenges and Emerging Technologies in Multiplexing LFAs

LFAs are membrane-based platforms typically used to qualitatively detect targeted analytes in complex samples within 5–30 mins [13]. LFAs are currently the only rapid diagnostic format that fully meets the World Health Organization (WHO)’s ASSURED (affordable, sensitive, specific, user-friendly, rapid and robust, equipment-free and deliverable to end users [14]) criteria for testing in resource-constrained settings. These qualities also have seen LFAs become ubiquitous in various settings including hospitals, public and private clinics and laboratories.

Despite decades of use, improvement of this format remains in its infancy. Significant efforts have been made at improving LFA sensitivity and specificity; however, the most coveted iteration of the LFA would be able to measure multiple biomarkers simultaneously [15]. Presently, few multiplexed LFAs exist in the market and the vast majority of these are developed to detect toxins, drugs and more general clinical biomarkers such as liver enzymes, cardiac and hematological markers. Multiplexing of infectious disease biomarkers has apparently been complicated by the inherent limitations in the LFA system and platform. LFAs predominantly use immobilized antibodies or proteins as capture ligands placed at specific locations (typically striped as a line) across a membrane, and in-solution detector molecules typically labeled with colloidal gold. While largely versatile, rapid, with potential for high accuracy, this system is also susceptible to drawbacks such as lot-to-lot variability, antibody cross-reactivity, and dependence on capillary action between sample and LFA strip components [2,16]. Any multiplexed formats utilizing traditional immunoassay principles would be expected to overcome the compounded technical challenges of single-biomarker LFAs in order to be useful and scalable [17].

The technical challenges of multiplexing LFAs have been extensively reviewed elsewhere [15,18], but the main concern appears to be potential cross-reactivity, which limits the number and types of biomarkers that can be combined. Cross-reactivity, which causes non-specific binding, higher background and/or false positives, has been observed for several infectious diseases such as flavivirus infections (dengue with zika virus) [19] and helminthic infections (strongyloidiasis with filariasis) [20], which incidentally may also have overlapping symptoms. Multiplexing several test lines on a single LF strip is also confined to physical limits, the number of conjugates, as well as the flow changes when passing through multiple lines. Arguably, these physical limitations may be circumvented using recently reported creative designs using multiple strips [21,22], spot arraying within the test field of the LF membrane, a pixelation technology currently commercially offered by Symbolics LLC [23], or optically encoded regions embedded within a porous membrane [17]. Further operational and quality assurance issues exist, primarily related to adapting available designs of assay evaluations (particularly the use of appropriate patient and control populations), methods of calibration, validation and assessment of diagnostic accuracy and reproducibility that have been established for single-biomarker LFAs [18]. The “simplicity” of LFAs that allow for minimal training may be compromised if multiple results need to be interpreted on a single test; however, this too may be addressed with the increasing availability of portable battery-operated instruments and smart devices. In addition, many good biomarkers that are available in single-test LFAs, are proprietary and/or patented, and maneuvering proprietary interests of different parties who may not be readily willing or able to share their technology may pose additional challenges.

From a research perspective, there have been encouraging reports of multiplexed LFA prototypes in recent literature. Notable mentions include (1) multiplexed LFA developed using multi-colored silver nanoparticles (AgNPs) to give colored test lines for simultaneous detection of dengue, yellow fever, and Ebola viruses [24]; (2) a novel disc design incorporating 10-dipsticks lined with 10 different foodborne pathogen biomarkers using up-converting phosphor (UCP) particle as the reporter for increased sensitivity and tolerance of sample interference [21]; (3) an LFA also utilizing the UCP technology to detect multiple cytokines in leprosy [25] and (4) an LFA-based multiplex for diagnosing acquired immune deficiency syndrome (AIDS), and hepatitis C and A (HAV, HCV) using proteinticle-based 3D probes that display different viral antigens [26].

Despite these developments in the literature, very few multiplexed LFAs have become commercial products [12], suggesting the existence of critical challenges that impede the translation of multiplexed LFAs beyond laboratory research scale. Table 1 briefly summarizes the key challenges in the path of producing a multiplexed LFA diagnostic product that can reach the end-user market, broadly categorized as (1) technical or assay development and (2) quality control and operational, and appraises whether there is scope to overcome these challenges with consideration of recent developments and emerging enabling technologies (Table 1).

## 3. Way Forward

From a technical perspective, the literature indicates that multiplexing on LFA may require capture and detector systems that move away from conventional monoclonal antibodies and proteins. While the examples provided focused on multiplexed LFAs for multiple diseases, we expect the challenges and opportunities discussed to be applicable for multiplexed LFAs designed to detect a panel of biomarkers for diagnosis of a single infectious disease. In particular, alternative biomolecules such as aptamers, recombinant antibodies, and engineered protein scaffolds are expected to have more capacity to overcome any potential cross-reactivity within and between assays combined on one multiplexed LFA test [26,27,28]. The critical issue here is whether there will be access to these alternative biomolecular reagents from commercial sources, particularly for LFA developers without collaborative links to research groups producing these reagents. For example, the development of aptamers through SELEX (systematic evolution of ligands by exponential enrichment) requires high initial investment and specialized skills. This limits the number of groups that can exploit this approach, and prevents developers from delving into multiplex LFA formats for detecting biomarkers. This is in contrast to the current scenario for developers using monoclonal antibodies, which are quality assured and produced against an array of targets available for order on a supplier’s catalogue, speeding up the process for diagnostic research and development. Hence, before multiplexed LFA can become a reality, these promising biomolecular alternatives first need to be accessible and reach the scale of mass production.

Similarly, many reports on multiplexed LFA in recent literature utilized alternative probes and detection systems that are more amenable to multiplexing with the support of readers [17,24,29,30]. Although ASSURED criteria recommend equipment-free POC diagnostic tests, portable battery-operated readers and smartphones with special applications are becoming more readily available to remove reliance on user-interpretation. Among the more promising examples is a low-cost cell phone-based dongle platform able to perform the functions of a reader solely requiring energy from the connected smartphone [31,32]. On top of facilitating interpretation of multiplexed LFA test results, these instruments have the capacity to collect data for analytical and monitoring purposes. Such data collection and monitoring would be even more critical to evaluate the performance of these more complex LFA tests in the field over time. The advent of Internet of things (IoT) further allows the test results to be quickly transmitted to the relevant agencies, especially for notifiable diseases. While this can be a positive development for monitoring and surveillance, it also brings forth new challenges such as regulatory requirements in the handling and safety of the data [12]. Regardless, the use of any form of reader is anticipated to increase the overall price of the test, which may be a contentious issue for adoption in resource-constrained settings. Without reliance on readers for interpretation of test results, multiplexed LFA may require creative designs [21,23], or alternative labeling methods such as using shapes or multicolored nanoparticles [24]. Either of these approaches will require a transdisciplinary approach, with key skillsets in biotechnology, chemical engineering, software/application programming, industrial design and collaboration with industry partners.

**Figure 1:**
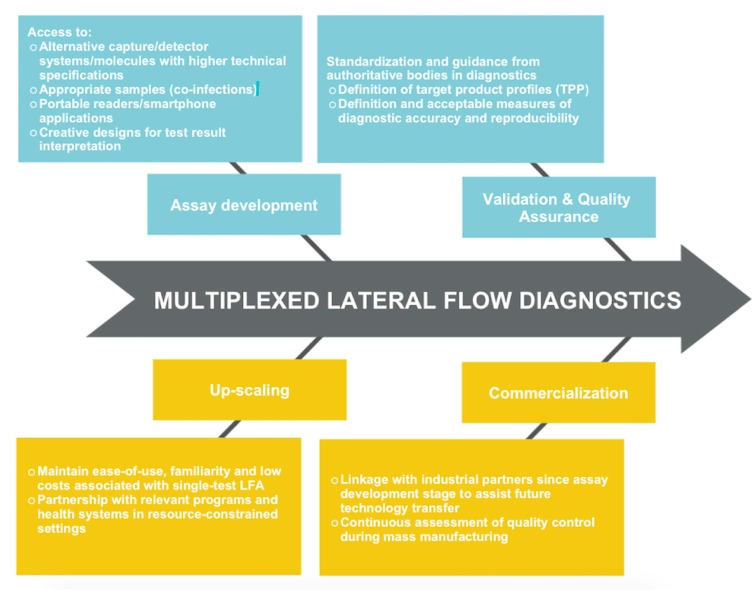
Flowchart of key factors in the path towards translationally viable multiplexed Lateral flow assays (LFA) diagnostics.

Arguably the most difficult hurdles to overcome lie in validation, commercial translation and quality assurance of any multiplex LFA device that shows promise in the laboratory or research scale. Furthermore, validation of diagnostic accuracy and reproducibility will require judicious study designs, and critically so, access to single-infection and co-infection patient samples and appropriate control samples. Where single-test assays could utilize the coefficient of variation (CV) (ratio of the standard deviation to the mean average) as a measure of intra- and inter-assay variability, it remains questionable whether the CV is still usable for multiplex tests, as well as the range of values of acceptable precision or inaccuracy. Validation studies of other platforms such as multiplex chemiluminescent immunoassays suggest that while intra-assay CVs for multiplex may be comparable with single ELISAs, inter-assay CVs indicate higher variability between the multiplex assays [33,34]. Recommendations recently exist for validation of multiplex tests in drug development [35], with considerations for construction of a biomarker work plan, the number of analytes in one test, and the alignment of statistical acceptability criteria with intended use, and clinical, biological, and epidemiological relevance of analyte(s). While these recommendations may be applicable for diagnostic applications, developers may rely on authoritative bodies in diagnostics such as the Foundation for Innovative New Diagnostics (FIND) and PATH and regulatory bodies such as the Food and Drug Administration (FDA) to address the many uncertainties with regards to ascertaining diagnostic accuracy and reproducibility. Figure 1 highlights the factors that may promote efforts towards successful development and translation of a multiplexed LFA.

## 4. Conclusions

In summary, the path towards a multiplexed diagnostic LFA that is deliverable and can benefit communities living in resource-constrained settings appears rife with challenges. However, recent developments in biotechnology have sparked hope that the tide may be changing, and an LFA with the ability to diagnose or rule out multiple infections on a single-test within one encounter at a peripheral health post, clinician’s office or emergency settings such as refugee camps may one day become a reality. To further facilitate the successful development of multiplex LFAs for diagnosis of infectious disease, access to alternative biomolecules, samples and enabling technology must first be more readily available to the wider community of LFA developers. Finally, the efforts of developers in the laboratories can be augmented with guidance from authoritative and regulatory bodies, and pathways for forming partnerships with the industry as well as with groups that will ultimately market, use, or advocate for use of a multiplexed LFA diagnostic test in resource-constrained settings.

## Figures and Tables

**Table 1:** Assessment of challenges in multiplexing lateral flow assays (LFA).

	Challenge	Difficulty *	Remarks	References (No.)
Technical and Assay Development	Cross-reactivity of immobilized capture and detector antibodies with non-targeted analytes limits multiplexing capability. Antibodies that have been validated on single-assays may be cross-reactive when multiplexed with other assays. Some proteins may not be usable due to nonspecific binding which may reduce assay sensitivity.	Possible	Aptamer oligonucleotides are reportedly cheaper than antibodies. Dual recognition element LFA (DRELFA) has been shown to overcome cross-reactivity of antibody and slow-binding kinetics of aptamers. Phage-display derived recombinant antibodies with potential for higher specificity. Proteinticles (genetically modified proteins) used as 3D probes demonstrate increasing sensitivity of multiplexed LFA for anti-viral detection in HIV, HAV, and HCV.	Lee, et al. [26] Le, et al. [27] Ch’ng, et al. [28]
	Physical limitation of LFA strip to only a few test lines placed at specific locations, the number of which will affect the test flow rate.	Possible	Pixelation technology for spot array commercially available to test developers since 2015. Parallume lanthanide optical encoding technology enables deep optical multiplexing using a companion reader, proof-of-concept demonstrated for multiplex detection of anti-HIV, anti-HCV etc. Multiple strips incorporated in disc design, allowing detection of 10 different biomarkers simultaneously.	O’Farrell, et al. [15] Haushalter, et al. [17] Zhao, et al. [21]
	Clinical specimens for assay development and test validation will require patients with multiple co-infections relevant to the test, which may be difficult to acquire.	Difficult-Possible	Available cohort studies monitoring and diagnosing several diseases relevant to the local epidemiology may be an important source of specimens with relevant co-infections.	
	Hook effect arising from an excess of unlabeled analytes competing with labeled analytes causes decrease in test signal for samples with high analyte concentration. Compounded in multiplexed assays with cross-reactivity.	Possible	With the use of a portable imaging devices, a reaction kinetics-based technique (example: C-reactive protein) has been proposed to significantly increase the dynamic range of LFAs, overcoming the problem of hook effect.	Rey, et al. [29]
**Operational and Quality Control**	Inter- and intra-assay variability (estimated by coefficient of variation (CV)) for multiplexed test are questionable and acceptable values of reproducibility in multiplexed tests undefined.	Difficult	Limited guidance/regulation available for development of multiplexed LFAs may require more concerted effort from authoritative diagnostic and regulatory bodies.	Ellington, et al. 2010 [18]
	Acceptable level of imprecision of LF assays undefined. Unclear whether failure of one test within the multiplex constitutes entire test failure.			
	Conventional LFAs with visual interpretation become complicated with increasing number of test results and corresponding controls, particularly with positive results with low signals.	Possible	Test results may be varied using different labels (e.g., multicolored silver particles), or structures/shapes. Portable battery-operated readers, smartphones with diagnostic applications or accessories (dongles) are becoming readily available to remove reliance on user-interpretation, and allows for alternative probes and also produce data for analytical and monitoring.	Yen, et. al. [24] Martinez-Hurtado, et. al., [30] Guo, et al. [31] Laksanasopin, et al. [32]

* Judgment on degree of challenge difficulty to overcome marked as Difficult or Possible with consideration of current developments, trends and new/alternative technologies/materials in the literature.
